# Hyperoxemia may be more beneficial for patients with sepsis

**DOI:** 10.1186/s13054-024-05017-8

**Published:** 2024-07-19

**Authors:** Liyuan Peng, Xiaoming Qin, Lvlin Chen

**Affiliations:** Department of Critical Care Medicine, Affiliated Hospital of Chengdu University, 82 North 2nd Section of 2nd Ring Road, Jinniu District, Chengdu, 610041 Sichuan China

To the Editor,

We have read with great interest the clinical study by Dong-gon et al. published in Critical Care [1]. The study showed a robust correlation between higher PaO_2_ (≥ 80 mmHg) during the first three ICU days and a lower 28-day mortality. The optimal PaO_2_ range represents an intriguing and significant subject for exploration.

Our department is the general Intensive Care Unit (ICU) of a tertiary teaching hospital, with sepsis-related patients making up approximately one-fourth to one-third of the total patient population. We are deeply interested in exploring the issue of "optimal oxygenation". Therefore, we conducted a statistical analysis of patients admitted to our department with sepsis.

We retrospectively examined adult patients who admitted to ICU with sepsis as the primary diagnosis. Data were collected from consecutive electronic health records of Affiliated Hospital of Chengdu University between January 2021 and December 2023. The inclusion criteria were as follows: (1) aged ≥ 18 years; (2) The primary diagnosis was related to sepsis; (3) the length of ICU stay ≥ 3 days. The exclusion criteria included a lack of data on PaO_2_ during the first three days of ICU admission due to missing information, less than three days of ICU stay, or readmission or the presence of severe concurrent organ failures (e.g., myocardial infarction, uremia, advanced malignant tumor).

We also defined ICU day 1 as the time from ICU admission to the first midnight, ICU day 2 as the next 24 h from the first midnight, and ICU day 3 as the time from the second midnight to the third midnight. The values of PaO_2_ over the first three days of ICU admission were collected. When multiple arterial blood gas analysis was performed, the lowest result regarding PaO_2_ was recorded. Based on the PaO_2_ value from arterial blood gas analysis, patients who maintained a PaO_2_ ≥ 80 mmHg during the first three days in the ICU were assigned to the liberal PaO_2_ group, while the remaining were included in the conservative PaO_2_ group.

We used univariate logistic regression analysis to examine the association between the PaO_2_ and the clinical outcomes (ICU mortality, in-hospital mortality and invasive ventilation). This analysis provided odds ratios and 95% CIs. Two-sided *P* values < 0.05 were considered significant. All analysis were performed using SPSS Statistics for Windows, version 24.0.

During the study period, 489 adult patients admitted to the ICU were screened, after 197 patients were excluded from the analysis (94 patients had an ICU stay of less than 3 days, 10 patients were readmitted, 93 patients had severe concurrent organ failures), ultimately 292 patients were included in the final analysis (Fig. 1).

**Fig. 1:**
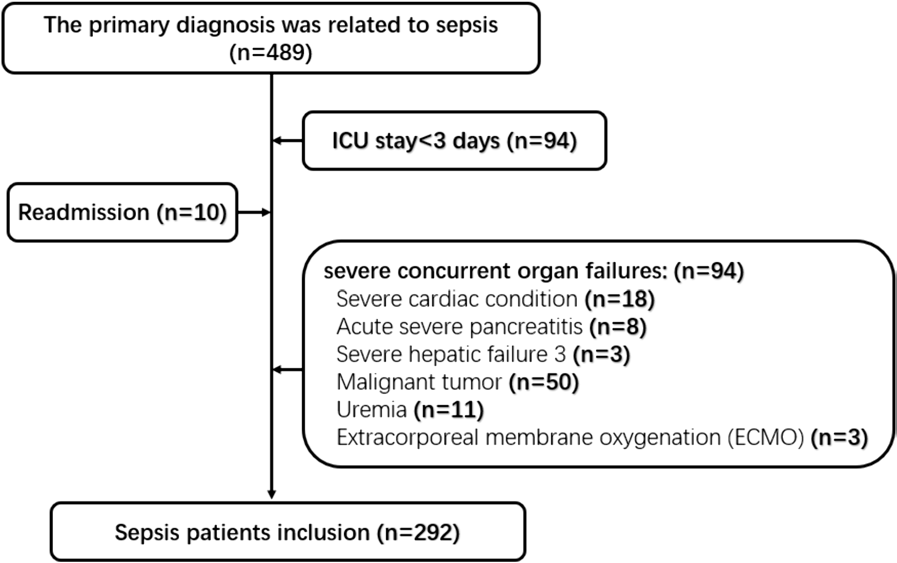
Flow chart

The total in-hospital mortality rate and ICU mortality for our study population were 35.3% (*n* = 103) and 32.5% (*n* = 95). In-hospital mortality and ICU mortality were significantly different between the conservative PaO_2_ group and the liberal PaO_2_ group. The liberal PaO_2_ group showed a significantly higher probability of survival (OR 0.53, 95% CI 0.32–0.88, for in-hospital mortality; OR 0.49, 95% CI 0.29–0.83, for ICU mortality). There was no statistical difference in invasive ventilation between the two groups (Table 1).

**Table 1:** Comparing outcomes between conservative PaO_2_ and liberal PaO_2_ for sepsis patients

Outcomes	Conservative PaO_2_	Liberal PaO_2_	OR	95% CI	*p*-value
ICU mortality, *n* (%)	66/170 (38.8)	29/122 (23.8)	0.49	0.29–0.83	0.007
Hospital mortality, *n* (%)	70/170 (41.2)	33/122 (27.0)	0.53	0.32–0.88	0.013
Invasive ventilation, *n* (%)	118/170 (69.4)	75/122 (61.5)	0.70	0.43–1.15	0.158

Our findings are consistent with Dong-gon’s [1], indicating that real-world data can support the results of their results. This finding is of great significance. Sepsis is at a high risk of morbidity and mortality, imposing a significant global economic burden. Currently, in line with various guidelines and those specific to COVID-19 [2, 3], it is recommended to maintain SpO_2_ levels below 100%. This is because exposure to hyperoxemia may cause oxidative damage, inflammation, and increase alveolar-capillary permeability [4–8]. The results of this study are poised to shift the perspectives of ICU doctors, benefiting a larger population of sepsis patients.

Certainly, obtaining more reliable results requires high-quality randomized controlled trials (RCTs) for confirmation. It is acknowledged that patients in extremely critical conditions may not reach a PaO_2_ level of at least 80 mmHg, resulting in increased mortality rates. Further research is essential to mitigate the influence of this scenario. Moreover, additional studies are needed to establish the optimal cutoff value for liberty PaO_2_ and to address inquiries concerning the duration of maintaining elevated PaO_2_ levels, all of which warrant further investigation.

In summary, according to the study’s data and our findings, we suggest, that sepsis patients was given higher PaO_2_(PaO_2_ ≥ 80 mmHg) for the first 3 ICU days. Overall oxygenation target for sepsis patients should be subject to further investigations.

## Data Availability

The datasets used and/or analyzed during the current study are available from the corresponding author on reasonable request.

## Abbreviations

ICU: Intensive care unit
PaO_2_: Partial pressure of oxygen in arterial blood
OR: Odds ratio
CI: Confidence interval
COVID-19: Corona virus disease-2019
RCTs: Randomized controlled trials
